# Differences in Mode Preferences, Response Rates, and Mode Effect Between Automated Email and Phone Survey Systems for Patients of Primary Care Practices: Cross-Sectional Study

**DOI:** 10.2196/21240

**Published:** 2021-01-11

**Authors:** Sharon Johnston, William Hogg, Sabrina T Wong, Fred Burge, Sandra Peterson

**Affiliations:** 1 Department of Family Medicine University of Ottawa Ottawa, ON Canada; 2 Department of Family Medicine Institu du Savoir, Hôpital Montfort University of Ottawa Ottawa, ON Canada; 3 Centre for Health Services and Policy Research University of British Columbia Vancouver, BC Canada; 4 Department of Family Medicine Dalhousie University Halifax, NS Canada

**Keywords:** response rates, primary care, mixed-mode survey

## Abstract

**Background:**

A growing number of health care practices are adopting software systems that link with their existing electronic medical records to generate outgoing phone calls, emails, or text notifications to patients for appointment reminders or practice updates. While practices are adopting this software technology for service notifications to patients, its use for collection of patient-reported measures is still nascent.

**Objective:**

This study assessed the mode preferences, response rates, and mode effect for a practice-based automated patient survey using phone and email modalities to patients of primary care practices.

**Methods:**

This cross-sectional study analyzed responses and respondent demographics for a short, fully automated, telephone or email patient survey sent to individuals within 72 hours of a visit to their regular primary care practice. Each survey consisted of 5 questions drawn from a larger study’s patient survey that all respondents completed in the waiting room at the time of their visit. Automated patient survey responses were linked to self-reported sociodemographic information provided on the waiting room survey including age, sex, reported income, and health status.

**Results:**

A total of 871 patients from 87 primary care practices in British Columbia, Ontario, and Nova Scotia, Canada, agreed to the automated patient survey and 470 patients (45.2%) completed all 5 questions on the automated survey. Email administration of the follow-up survey was preferred over phone-based administration, except among patients aged 75 years and older (*P*<.001). Overall, response rates for those who selected an emailed survey (369/606, 60.9%) were higher (*P*<.001) than those who selected the phone survey (101/265, 38.1%). This held true irrespective of age, sex, or chronic disease status of individuals. Response rates were also higher for email (range 57.4% [58/101] to 66.3% [108/163]) compared with phone surveys (range 36% [23/64] to 43% [10/23]) for all income groups except the lowest income quintile, which had similar response rates (email: 29/63, 46%; phone: 23/50, 46%) for phone and email modes. We observed moderate (range 64.6% [62/96] to 78.8% [282/358]) agreement between waiting room survey responses and those obtained in the follow-up automated survey. However, overall agreement in responses was poor (range 45.3% [43/95] to 46.2% [43/93]) for 2 questions relating to care coordination.

**Conclusions:**

An automated practice-based patient experience survey achieved significantly different response rates between phone and email and increased response rates for email as income group rose. Potential mode effects for the different survey modalities may limit multimodal survey approaches. An automated minimal burden patient survey could facilitate the integration of patient-reported outcomes into care planning and service organization, supporting the move of our primary care practices toward a more responsive, patient-centered, continual learning system. However, practices must be attentive to furthering inequities in health care by underrepresenting the experience of certain groups in decision making based on the reach of different survey modes.

## Introduction

The development of an information infrastructure to support a learning health system in primary care has advanced significantly with the application of advanced analytics applied to data from electronic medical records and routinely collected administrative data [1]. However, in Canada most primary care is delivered in small community-based practices and, unlike the United Kingdom, there is no national or provincial infrastructure to measure and report patient experience data for primary care. Such data collection remains logistically challenging and relatively expensive for smaller practices [2]. While waiting room surveys often provide good response rates, they are costly, burdensome to practices, introduce a sampling bias toward older and more complex patients, and are limited to patients who physically attend a practice [1,3].

A growing number [4] of health care practices are adopting software systems [5] that link with their existing electronic medical records to generate outgoing phone calls, emails, or text notifications to patients for appointment reminders or practice updates. While practices are adopting this software technology for service notifications to patients, it is not clear whether such an approach would be acceptable to survey a practice’s patients on experience or outcome measures selected by the practice to advance their quality improvement efforts. The data on response rates for electronic surveys in primary care is rudimentary compared with that for hospital surveys [4], but response rates of 20% to 30% [6,7] have been found recently for emailed surveys linked to primary care practices [6-8]. The objective of this study is to assess the mode preferences, response rates, and mode effect for a practice-based automated patient survey using phone and email modalities to patients of primary care practices.

## Methods

### Study Sample

This cross-sectional study analyzed mode preferences, response rates, and respondent demographics for a short, fully automated, telephone or email patient survey to consenting individuals who had recently attended their regular primary care practice. Within our larger study, Transforming Community-Based Primary Health Care Delivery through Comprehensive Performance Measurement and Reporting (TRANSFORMATION), patients from 87 primary care practices in British Columbia, Ontario, and Nova Scotia, Canada, were asked to complete a waiting room survey between 2014 and 2016. The automated patient survey system was tested on a convenience sample of those participants who consented to receiving an additional postvisit survey by email or phone. Eligible patients had to speak English or French and have a valid telephone number or email address. Patients were asked to specify their preferred contact modality, phone or email, and provide their name and contact information to an on-site research team member.

The contact information and unique identifying number for consenting patients was entered manually by survey administrators and uploaded to a cloud-based server via a software console. Upon receipt of the information, the administering information technology company collaborator, Cliniconex [9], programmed the appropriate survey mode and language (English or French) and randomly assigned the order of 5 survey questions. Once the survey was administered by Cliniconex, all contact information was deleted, and only the unique identifying number was retained on the server.

### Survey Administration

Participants received an automated phone or email survey within 72 hours of visiting the practice. A phone survey response was recorded as completed only if the patient could be reached at the phone number on file, accepted the call, and completed all 5 survey questions. The phone survey was initially attempted twice, and then registered as incomplete if no answer was received. Partway through the study, the number of attempts was increased (4 call attempts) to facilitate higher response rates. For those who chose an email survey mode, an email was sent once containing the introduction and a web link to the survey. An email survey was recorded as complete if all 5 survey questions were answered.

Each survey consisted of 5 questions drawn from the TRANSFORMATION study’s waiting room patient survey [10]. The questions were selected to relate to patients’ experience with primary care and/or their practice. Two question prompts were modified from their original form in the paper waiting room survey to reflect the timing of the survey administration. When administered in the waiting room, questions 1 and 2 were prompted with “After seeing the family doctor or nurse today...”; on the automated patient survey, patients were prompted with “At your last visit with your family doctor or nurse practitioner....” See Multimedia Appendix 1 for the wording of the survey questions in the paper waiting room survey and the postvisit automated survey. Phone survey responses were stored in a secure password-protected site on a secure server. Email responses were sent directly to a hospital-based server and managed using Research Electronic Data Capture tools [11].

The unique identifying numbers were used to link automated patient survey responses to self-reported sociodemographic information on the paper waiting room survey, completed during the participant’s visit to their practice, including age, sex, reported income, and health status.

### Data Analysis

To detect any response bias inherent in using an automated email or phone survey system, we used Pearson chi-square tests to compare the sociodemographic profile of those who completed the automated patient survey (responders) with those who did not complete the automated patient survey (nonresponders). The comparison group of nonresponders contained those who either participated in the paper waiting room survey but refused the automated survey or agreed to the automated survey but did not complete all 5 questions. We also conducted Wilcoxon rank-sum tests on the paper waiting room survey responses, comparing differences in mean responses between those who completed the automated patient survey and those who did not. We conducted chi-square tests to compare automated patient survey mode preference (email or phone) and response rates both across and between patient sociodemographics. A Cochran-Armitage test for trends was also used to examine variation in mode preference by age and income. All analyses were performed using SAS software version 9.4 (SAS Institute Inc).

The primary outcome measure response rate was pooled across all practices as we were interested in differences across dependent variables of age and attributed socioeconomic status rather than regional variations.

To identify a potential mode effect, secondary analyses explored responses for each question across the 3 survey modes, email and phone (automated patient survey) and paper (waiting room). Test-retest analysis was undertaken, comparing each patient’s responses from the waiting room survey to their responses to the corresponding automated survey question. The percentage of concordant and discordant responses were determined by comparing waiting room derived responses with those from subsequent survey data. Weighted kappas were calculated to compare this concordance in survey responses by survey mode. Mean responses were also compared (using Wilcoxon signed-rank test) across responses to the corresponding questions from the paper waiting room survey and the automated patient survey (total and by mode).

This study was approved by the behavioral research ethics boards at Fraser Health (RHREB 2015-017), University of British Columbia (H13-01237), Ottawa Health Science Network (20140485-01H), Bruyère Continuing Care (M16-14-029), and the Nova Scotia Health Authority (CDHA-RS/2015-150).

## Results

### Response Bias

Of those who agreed to the automated patient survey, 69.6% (606/871) of participants chose to receive the survey by email compared with telephone. This group represented 45.15% (871/1929) of the participants who initially consented to completing a paper waiting room survey (Table 1). Of those who agreed to the survey, 55.6% (484/871) responded and 97.1% (470/484) completed all 5 questions (24.36% [470/1929] of those who completed the paper waiting room survey and 54.0% [470/871] of those who agreed to the automated patient survey). Respondents to the automated patient survey tended to be older, were more likely to be women, had higher income, and reported a larger number of chronic conditions than those not completing the survey. There was no significant difference in paper waiting room survey responses between those who completed the automated patient survey and those who did not (Table 2).

**Table 1:** Comparison of those who completed the automated patient survey to those who did not^a^.

Characteristics		Total	Completed automated patient survey			Did not complete automated patient survey^b^
			Phone	Email	Total^c^	
Consented to automated patient survey, n (%)		871 (45.2)	N/A^d^	N/A	N/A	N/A
Automated patient survey response rate, n (%)		484 (55.6)	N/A	N/A	N/A	N/A
Automated patient survey completion rate (all 5 questions), n (%)		470 (54.0)	N/A	N/A	N/A	N/A
Overall, n (%)		1929	97 (5.0)	361 (18.7)	458 (23.7)	1471 (76.3)
**Age group, n (%)**						
	18-24	99	0 (0)	11 (3.0)	11 (2.4)	88 (6.0)
	25-64	1251	50 (51.5)	263 (72.9)	313 (68.3)	938 (64.2)
	65-74	377	35 (36.1)	62 (17.2)	97 (21.2)	280 (19.2)
	75+	191	12 (12.4)	25 (6.9)	37 (8.1)	154 (10.5)
	χ^2^ *P* value^e^	N/A	<.001	.006	.006	N/A
	Cochran-Armitage 2-sided *P* value^f^	N/A	<.001	.14	.86	N/A
**Sex, n (%)**						
	Male	634	28 (29.2)	97 (27.0)	125 (27.5)	509 (35.3)
	Female	1263	68 (70.8)	262 (73.0)	330 (72.5)	933 (64.7)
	χ^2^ *P* value^e^	N/A	.22	.003	.002	N/A
**Income ($), n (%)**						
	<20,000	266	23 (25.6)	29 (8.5)	52 (12.0)	214 (17.0)
	20,000-40,000	367	23 (25.6)	58 (17.0)	81 (18.8)	286 (22.7)
	40,000-60,000	340	18 (20.0)	74 (21.6)	92 (21.3)	248 (19.7)
	60,000-100,000	435	16 (17.8)	108 (31.6)	124 (28.7)	311 (24.7)
	>100,000	283	10 (11.1)	73 (21.3)	83 (19.2)	200 (15.9)
	χ^2^ *P* value	N/A	.15	<.001	.02	N/A
	Cochran-Armitage 2-sided *P* value^f^	N/A	.01	<.001	<.001	N/A
**Chronic conditions, n (%)**						
	0-1	637	12 (12.4)	115 (31.9)	127 (27.8)	510 (36.1)
	2	291	14 (14.4)	48 (13.3)	62 (13.6)	229 (16.2)
	3+	943	71 (73.2)	197 (54.7)	268 (58.6)	675 (47.7)
	χ^2^ *P* value	N/A	<.001	.06	<.001	N/A

^a^Total counts within categories vary due to missing data.

^b^Includes those who did not consent to having their automated patient survey linked to their waiting room survey responses.

^c^Excludes those who did not consent to having their automated patient survey linked to their waiting room survey responses.

^d^N/A: not applicable.

^e^χ^2^*P* value is comparing the distribution of the subgroup (eg, age, gender) between completed and not completed, for each mode and overall.

^f^Cochrane-Armitage *P* value for the presence of linear trend in proportions of completed and not completed across ordinal subcategories, for each mode and overall.

**Table 2:** Comparison of waiting room survey responses between those who completed the automated patient survey to those who did not^a^.

Waiting room survey questions	Response for those who also completed automated patient survey^b^, mean (SD)	Response for those who did not complete automated patient survey^c^, mean (SD)	*P* value^d^
Given enough time	4.67 (0.60)	4.63 (0.75)	.88
Explained tests and treatments	4.70 (0.61)	4.66 (0.71)	.85
Told about potential side effects of medications	2.64 (0.60)	2.69 (0.55)	.30
Times when provider didn’t have access to recent tests or exam results	1.26 (0.51)	1.25 (0.52)	.63
Times when provider didn’t know about changes in treatment plan that another person recommended	1.23 (0.47)	1.20 (0.44)	.37

^a^Total counts within categories vary due to missing data.

^b^Excludes those who did not consent to having their automated patient survey linked to their waiting room survey responses.

^c^Includes those who did not consent to having their automated patient survey linked to their waiting room survey responses.

^d^Wilcoxon rank-sum test *P* value is comparing paper waiting room survey responses between completed and not completed.

### Response Rates

In this sample, email administration of the follow-up survey was preferred over phone-based administration, except among patients aged 75 years and older (Table 3). Among those who answered the automated patient survey, 97.1% (470/484) completed of all 5 questions. Thus, response rates include only those who answered all 5 questions. Overall, response rates for those who selected an emailed survey (369/606, 60.9%) were higher than those who received the phone survey (101/265, 38.1%). This held true irrespective of the age, sex, or chronic disease status of individuals. Response rates were also higher for email compared with phone surveys for all income groups except the lowest income quintile, which had similar response rates for phone and email modes. There was variation in response rates within email mode, with higher responses among more affluent individuals.

**Table 3:** Mode preference and response rates by subgroups.

Characteristic		Mode preference (n=871)				Completed automated patient survey (n=470)			
		Total, n (%)	Phone, n (%)	Email, n (%)	χ^2^ *P* value^a^	Total, n (%)	Phone, n (%)	Email, n (%)	χ^2^ *P* value^b^
**Age group, years**		N/A^c^	N/A	N/A	<.001^d^	N/A	N/A	N/A	N/A
	18-24	29 (3.4)	s^e^	26 (89.7)	N/A	11 (37.9)	0 (0)	11 (42.3)	.15
	25-64	553 (65.0)	133 (24.1)	420 (75.9)	N/A	313 (56.6)	50 (37.6)	263 (62.6)	<.001
	65-74	185 (21.7)	78 (42.2)	107 (57.8)	N/A	97 (52.4)	35 (44.9)	62 (57.9)	.08
	75+	84 (9.9)	45 (53.6)	39 (46.4)	N/A	37 (44.0)	12 (26.7)	25,871 (64.1)	<.001
	χ^2^ *P* value^f^	N/A	N/A	N/A	N/A	.045	.11	.18	N/A
	Cochran-Armitage 2-sided *P* value^g^	N/A	N/A	N/A	N/A	.16	.64	.60	N/A
**Sex**		N/A	N/A	N/A	<.001	N/A	N/A	N/A	N/A
	Male	262 (31.1)	101 (38.5)	161 (61.5)	N/A	125 (47.7)	28 (27.7)	97 (60.2)	<.001
	Female	581 (68.9)	154 (26.5)	427 (73.5)	N/A	330 (56.8)	68 (44.2)	262 (61.4)	<.001
	χ^2^ *P* value	N/A	N/A	N/A	N/A	.01	.008	.81	N/A
**Income ($)**		N/A	N/A	N/A	<.001^d^	N/A	N/A	N/A	N/A
	<20,000	113 (14.5)	50 (44.2)	63 (55.8)	N/A	52 (46.0)	23 (46.0)	29 (46.0)	>.99
	20,000-40,000	165 (21.1)	64 (38.8)	101 (61.2)	N/A	81 (49.1)	23 (35.9)	58 (57.4)	.007
	40,000-60,000	162 (20.7)	47 (29.0)	115 (71.0)	N/A	92 (56.8)	18 (38.3)	74 (64.3)	.002
	60,000-100,000	206 (26.3)	43 (20.9)	163 (79.1)	N/A	124 (60.2)	16 (37.2)	108 (66.3)	<.001
	100,000+	136 (17.4)	23 (16.9)	113 (83.1)	N/A	83 (61.0)	10 (43.5)	73 (64.6)	.06
	χ^2^ *P* value	N/A	N/A	N/A	N/A	.03	.83	.049	N/A
	Cochran-Armitage 2-sided *P* value	N/A	N/A	N/A	N/A	.002	.73	.01	N/A
**Income ($)**		N/A	N/A	N/A	<.001	N/A	N/A	N/A	N/A
	<20,000	113 (14.5)	50 (44.2)	63 (55.8)	N/A	52 (46.0)	23 (46.0)	29 (46.0)	NS >.99
	20,000+	669 (85.5)	177 (26.5)	492 (73.5)	N/A	380 (56.8)	67 (37.9)	313 (63.6)	<.001
	χ^2^ *P* value	N/A	N/A	N/A	N/A	.03	.30	.007	N/A
**# Chronic conditions**		N/A	N/A	N/A	<.001	N/A	N/A	N/A	N/A
	0-1	242 (28.7)	43 (17.8)	199 (82.2)	N/A	127 (52.5)	12 (27.9)	115 (57.8)	<.001
	2	126 (14.9)	42 (33.3)	84 (66.7)	N/A	62 (49.2)	14 (33.3)	48 (57.1)	.01
	3+	475 (56.4)	170 (35.8)	305 (64.2)	N/A	268 (56.4)	71 (41.8)	197 (64.6)	<.001
	χ^2^ *P* value	N/A	N/A	N/A	N/A	.29	.20	.22	N/A

^a^Comparing percentage distribution of mode preference across subgroups.

^b^Comparing percentage completed across subgroups.

^c^N/A: not applicable.

^d^Cochran-Armitage test for trend also gives *P*<.001.

^e^Indicates suppressed due to cell count less than 5.

^f^Comparing completion rates between subgroups (overall or by mode).

^g^Cochran-Armitage conducted to test for trends.

### Mode Effect

We observed moderate agreement between waiting room survey responses and those obtained in the follow-up automated survey (see Multimedia Appendix 2). However, overall agreement in responses was poor for 2 questions relating to care coordination. Among phone respondents, agreement in responses was generally poor, and phone responders were particularly critical with respect to care coordination (Table 4). Agreement between waiting room responses and subsequent email survey regarding interpersonal aspects of care was moderate and poor for items relating to care coordination.

**Table 4:** Comparison of responses to paper waiting room surveys and automated surveys among those who completed the automated patient survey.

Description	Waiting room response, mean (SD)	Automated patient survey response—by mode			
		Phone, mean^a^ (SD)	*P* value^b,c^	Email, mean^d^ (SD)	*P* value^c,e^
Given enough time (range 1-5^f^)	4.67 (0.60)	4.66 (0.72)	.15	4.67 (0.65)	.27
Explained tests and treatments (range 1-5^f^)	4.70 (0.61)	4.42 (0.97)	.04	4.57 (0.79)	<.001
Told about potential side effects of medications (range 1-3^g^)	2.64 (0.60)	2.63 (0.70)	>.99	2.68 (0.68)	.28
Times when provider didn’t have access to recent tests or exam results (range 1-3^h^)	1.26 (0.51)	1.97 (0.90)	<.001	1.65 (0.79)	<.001
Times when provider didn’t know about changes in treatment plan that another person recommended (range 1-3^h^)	1.23 (0.47)	1.94 (0.88)	<.001	1.52 (0.71)	<.001

^a^The final N available for analysis for each question varies slightly due to nonresponse (or not applicable choice). Mean responses are calculated only for those who answered both versions of the questions. Ns for each question for those who completed the automated patient survey by phone are as follows: Q1: 96; Q2: 96; Q3: 92; Q4: 93.

^b^Comparing phone automated patient survey response to waiting room response.

^c^Wilcoxon signed-rank test.

^d^The final N available for analysis for each question varies slightly due to nonresponse (or not applicable choice). Mean responses and paired mean differences are calculated only for those who answered both versions of the questions. Ns for each question for those who completed the automated patient survey by email are as follows: Q1: 358; Q2: 357; Q3: 290; Q4: 336; Q5: 360.

^e^Comparing email automated patient survey response to waiting room response.

^f^1=very poor; 5=very good.

^g^1=no; 3=yes, often, or always.

^h^1=never or rarely; 3=often or very often.

## Discussion

### Principal Findings

We successfully deployed an automated multimodal practice-based patient survey in 87 primary care practices. Overall, patient preference for the email survey mode was demonstrated; however, this was modified by age group and socioeconomic status. Indeed, completion rates for email were higher than most health care automated surveys [8] versus comparable response rates in the total sample [6]. However, it is unclear whether the lower consent rate (45.2%) from the total patient sample reflects lack of acceptability of an automated low-burden survey or survey fatigue among participants who had already completed a long waiting room survey. Despite this, the relatively high completion rate to the short email survey suggests this is a feasible and acceptable approach to collect patient reported data.

Our results show that the lowest income group had the lowest preference for the email mode and lowest response rate for the email survey while having the highest response rates for the phone survey. Our finding of the email responders being more likely to be female and of higher income echoes the pattern of a recent practice-based single-site email survey in Ontario [6]. A move to use email surveys to collect patient experience data would need to carefully monitor underrepresentation by lowest income groups to not exacerbate inequities in health care. The survey software, as it is used currently for appointment reminders, is usually deployed after linking with the electronic medical record to use patient contact information, so it is possible for automated surveys to track information such as approximate income based on postal code and oversample a population found to be underrepresented in responses.

Opportunities to match surveys to reported language preferences and the capacity to reach people by phone or email who do not frequently attend a practice or have a stable home address raises the potential for an automated survey to be particularly valuable in understanding the experience of some of the most vulnerable members of a practice population. However, there are still inequities in access to the internet, with lower income individuals and people living in rural areas having lower access [12-14]. Text messaging might be preferable to phone for some patients and increase the reach across sociodemographic groups.

The low concordance rate of responses on questions of care coordination between paper and automated survey, especially the phone survey, raises important questions about a mode effect and/or the role of true anonymity in responding to questions about one’s health care provider or practice in a waiting room compared with online or automated phone response. It is also possible that the paper survey questions on care coordination sensitized participants to the issue, and after their visit, they were more aware of breakdowns in optimal care, accounting for their more negative responses with the automated survey following their practice visit. Additionally, the care coordination questions had negative phrasing, which may have been more confusing for phone responders.

Cost-effectiveness was not the focus of this study. However, at two-thirds the completion rate compared with email, a phone survey would cost one and a half times as much. The cost of deploying a tailored automated patient outreach message and linked survey from the software company we collaborated with includes a 1-time practice start-up fee of $500 CAN (US $390) and an annual per-provider fee of $600 CAN (US $468). For an average practice of 4 providers and 5200 patients, an email survey would cost about 25 cents (US 20 cents) if each patient were sent a message and survey twice per year or less than 15 cents (US 12 cents) if most patients were sent a survey 4 times per year. Higher response rates make the approach more cost-effective for the email mode since automated systems frequently charge per survey sent. For quality improvement data collection, practices would not need to seek prior consent to contact patients. However, efforts to enhance patient buy-in and achieve higher response rates would be key to the cost-effectiveness of this approach. As practices seek better ways to engage patients and collect patient-reported experience measures and patient-reported outcome measures, it is essential to be sensitive to the response burden on patients and promote a culture in which patients understand the purpose of surveys and feel their insights and time are valued [15]. This may help build a partnership with patients in practice-based surveying as a way to give patients more influence in the system and their care.

The capacity of this proposed system to link collection of patient-reported measures with clinical services, such as appointment reminders or preventive care reminders, could improve the response rates received on general surveys of patient experiences, improving quality and reducing costs [2]. Such an approach would have the benefit of being able to deploy surveys to all patients or ones with prespecified criteria (eg, people who just attended the practice, have not attended in over a year, have a recent hospital discharge). Such a survey could be linked with data automatically extracted from electronic medical records or a registry developed by providers, offering an even greater opportunity to understand patient experiences and outcomes. Additionally, an automated system can spread the burden of response across a wide and/or randomly selected segment of a practice’s patient population, asking different questions to different patients on an ongoing or rolling basis, enhancing reach and reducing cost compared with traditional waiting room surveys.

Increasingly, electronic medical records are being used to collect patient-reported outcome measures that are inputted directly into the patients’ chart. This approach offers the benefit of supporting a patients’ immediate care. However, this approach creates a burden for the provider or practice to review data automatically put into a patient chart in a timely manner. Keeping a patient automated survey function distinct from clinical care may be attractive to providers and practices who need to manage their workflow and feel overburdened with data and data requests already.

As a survey method, an automated patient survey offers some attractive features. Response rates and sample bias can be easily calculated for parameters such as age, gender or income as estimated by postal code without adding to patient burden of filling this information in. Based on continually updated information on filled surveys, ongoing distribution (sampling) parameters can be set to minimize or account for any bias that may arise. Automated surveys can be deployed at regular intervals determined by the practice and would not burden practice staff, providers, or even patients during a visit, thus avoiding interruptions or additional work.

As more practices are collecting email addresses from their patients and patients expect email communication options, an automated patient engagement system with an embedded survey is feasible. Practices already using this or a similar technology to serve patients through outreach reminders may be more willing to participate in data collection initiatives that use this same infrastructure for quality improvement or research.

### Limitations

There are some limitations to consider in interpreting the findings of this study. Initial recruitment into the TRANSFORMATION study was through a convenience sample of patients from primary care practices across British Columbia, Ontario, and Nova Scotia. As such, patients who were recruited into the study may not be representative of patients generally across Canada, potentially limiting generalizability. Additionally, potential for selection bias is further compounded by relatively low overall response rates by participants of the automated patient survey, who were recruited from the initial convenience sample of patients enrolled into the larger study.

### Conclusions

An automated practice-based patient experience survey achieved significantly different response rates between phone and email and increased response rates for email as income group rose. The higher response rates of the email surveys make a phone approach less cost-effective. However, care must be paid to furthering inequities in health care by underrepresenting the experience of certain groups in decision making. Further, potential mode effects for the different survey modalities may limit multimodal survey approaches.

An automated communication system will become even more valuable as the stock of high-quality and validated instruments to measure patient-reported outcomes grows over the next decade [16]. An automated system that enables targeted outreach surveys with minimal burden on patients and providers could facilitate the integration of patient-reported outcomes into care planning and service organization, supporting the move of our primary care practices toward a more responsive, patient-centered, continual learning system.

## Appendix

Multimedia Appendix 1 Survey questions and response options for the automated patient surveys and paper waiting room surveys.

Multimedia Appendix 2 Concordance of automated patient survey responses compared with paper waiting room survey responses.

## Abbreviations

TRANSFORMATION: Transforming Community-Based Primary Health Care Delivery Through Comprehensive Performance Measurement and Reporting
