# Supplementary material for: Differences in Mode Preferences, Response Rates, and Mode Effect Between Automated Email and Phone Survey Systems for Patients of Primary Care Practices: Cross-Sectional Study
Source: J Med Internet Res. 2021 Jan 11;23(1):e21240. doi: 10.2196/21240 (PMC7834947; doi:10.2196/21240)
Supplement: Multimedia Appendix 1 [file jmir_v23i1e21240_app1.docx]

Multimedia Appendix

| **Appendix A.** Survey questions and response options for the APS and Waiting Room Paper Surveys. | | |
| --- | --- | --- |
| **Description** | **APS Survey** | **Waiting Room Paper Survey** |
| **Given enough time** | | |
| Survey question | At your last visit with your family doctor or nurse practitioner, how good was the doctor or nurse at giving you enough time on a scale from 1 to 5, where 1 is very poor and 5 is very good | (Today) How good was the family doctor or nurse at giving you enough time? |
| Response options | 1= very poor | 1=Very poor |
|  | 2 | 2=Poor |
|  | 3 | 3=Fair – neither good nor poor |
|  | 4 | 4=Good |
|  | 5=very good | 5=Very good |
| **Explained tests and treatments** | | |
| Survey question | At your last visit with your family doctor or nurse practitioner, how good was the doctor or nurse at explaining tests and treatments on a scale from 1 to 5, where 1 is very poor and 5 is very good? | (Today) How good was the family doctor or nurse at explaining tests and treatments? |
| Response options | 1= very poor | 1=Very poor |
|  | 2 | 2=Poor |
|  | 3 | 3=Fair – neither good nor poor |
|  | 4 | 4=Good |
|  | 5=very good | 5=Very good |
| **Told about potential side effects from medications** | | |
| Survey question | Does your family doctor or nurse tell you about side effects you might get from a medicine on a scale from 1 to 5 where 1 is never and 5 is often or always? Press[IF PHONE]/Choose [IF EMAIL] 6 if you do not take any medicines. | (Past 12 months) Does your family doctor or nurse tell you about side effects you might get from a medicine? |
| Response options | 1=never | 1=No |
|  | 2 |  |
|  | 3 | 2=Yes, sometimes |
|  | 4 | 3=Yes, often or always |
|  | 5=often or always |  |
|  | 6=do not take any medicines | 4=I don’t take any medicines |
| **Times when provider didn't have access to recent tests or exam results** | | |
| Survey question | Thinking about the care you received from ALL the persons you saw in ALL the places you received care over the past 12 months, were there times when the person you were seeing did not have access to your recent tests or exam results on a scale from 1 to 3 where 1 is never or rarely and 3 is often or very often? | (All persons/all places, Past 12 months) Were there times when the person you were seeing did not have access to your recent tests or exam results? |
| Response options | 1=never or rarely | 1=Never or rarely |
|  | 2 | 2=Sometimes |
|  | 3=often or very often | 3=Often or very often |
|  | Missing/not given as an option | 4=Do not think this person needs to have this access |
| **Times when provider didn't know about changes in treatment plan that another person recommended** | | |
| Survey question | Thinking about the care you received from ALL the persons you saw in ALL the places you received care over the past 12 months, were there times when the person you were seeing did not know about changes in your treatment plan that another person recommended on a scale from 1 to 3, where 1 is never or rarely and 3 is often or very often? | (All persons/all places, Past 12 months) Were there times when the person you were seeing did not know about changes in your treatment that another person recommended? |
| Response options | 1=never or rarely | 1=Never or rarely |
|  | 2 | 2=Sometimes |
|  | 3=often or very often | 3=Often or very often |

This is a Multimedia Appendix to a full manuscript published in the J Med Internet Res. For full copyright and citation information see http://dx.doi.org/10.2196/jmir.xxxx
