# Supplementary material for: Differences in Mode Preferences, Response Rates, and Mode Effect Between Automated Email and Phone Survey Systems for Patients of Primary Care Practices: Cross-Sectional Study
Source: J Med Internet Res. 2021 Jan 11;23(1):e21240. doi: 10.2196/21240 (PMC7834947; doi:10.2196/21240)
Supplement: Multimedia Appendix 2 [file jmir_v23i1e21240_app2.docx]

Multimedia Appendix 2

| **Appendix B.** Concordance of APS responses compared to paper waiting room survey responses. | | | | | | | | |
| --- | --- | --- | --- | --- | --- | --- | --- | --- |
|  |  | **Phone** | | | **Email** | | |  |
| Description | | Concordant % | Answered more negatively % | Answered more positively % | Concordant % | Answered more negatively % | Answered more positively % | Chisq  *P*-value |
| Given enough time | | 64.6 | 10.0 | 25.0 | 78.8 | 12.0 | 9.2 | <.001 |
|  | Weighted kappa^a^ | 0.24 |  |  | 0.61 |  |  |  |
| Explained tests and treatments | | 70.8 | 19.8 | 9.4 | 73.4 | 19.6 | 7.0 | .73 |
|  | Weighted kappa^a^ | 0.27 |  |  | 0.52 |  |  |  |
| Told about potential side effects from medications | | 69.6 | 15.2 | 15.2 | 73.4 | 10.3 | 16.2 | .44 |
|  | Weighted kappa^a^ | 0.44 |  |  | 0.56 |  |  |  |
| Times when provider didn't have access to recent tests or exam results | | 46.2 | 45.2 | 8.6 | 60.1 | 32.4 | 7.4 | .05 |
|  | Weighted kappa^a^ | 0.10 |  |  | 0.12 |  |  |  |
| Times when provider didn't know about changes in treatment plan that another person recommended | | 45.3 | 44.2 | 10.5 | 66.1 | 28.3 | 5.6 | <.001 |
|  | Weighted kappa^a^ | 0.10 |  |  | 0.16 |  |  |  |
| ^a^Quadratic weights, Fleiss-Cohen method; ^b^ more negative responses reflect poorer assessment of performance and more positive reflect better assessment of performance | | | | | | | | |
